# COVID-19-specific Prefectural Hospital Bed Utilization Rate and In-hospital Mortality Among COVID-19 Patients Throughout the First 3 Years of the Pandemic in Japan

**DOI:** 10.2188/jea.JE20240395

**Published:** 2025-09-05

**Authors:** Hitomi Kimura, Mariko Hosozawa, Yuta Taniguchi, Kazumasa Yamagishi, Koji Kitajima, Mari Terada, Yusuke Asai, Norio Ohmagari, Hiroyasu Iso

**Affiliations:** 1 Institute for Global Health Policy Research, Bureau of Global Health Cooperation, Japan Institute for Health Security, Tokyo, Japan; 2 Doctoral Program in Medical Sciences, Graduate School of Comprehensive Human Sciences, University of Tsukuba, Ibaraki, Japan; 3 Department of Health Services Research, Institute of Medicine, University of Tsukuba, Ibaraki, Japan; 4 Department of Public Health Medicine, Institute of Medicine, and Health Services Research and Development Center, University of Tsukuba, Ibaraki, Japan; 5 Department of Public Health, Juntendo University Graduate School of Medicine, Tokyo, Japan; 6 Center for Clinical Sciences, Japan Institute for Health Security, Tokyo, Japan; 7 Disease Control and Prevention Center, Japan Institute for Health Security, Tokyo, Japan; 8 AMR Clinical Reference Center, Japan Institute for Health Security, Tokyo, Japan

**Keywords:** COVID-19, bed occupancy, mortality

## Abstract

**Background:**

We examined the association between the coronavirus disease 2019 (COVID-19)-specific prefectural bed utilization rate and in-hospital mortality during the first 3 years of the pandemic in Japan.

**Methods:**

This nationwide study included 58,175 COVID-19 patients from the COVID-19 Registry Japan, hospitalized between May 1, 2020 and November 30, 2022. Based on the weekly COVID-19-specific bed utilization rate in each prefecture at diagnosis, patients were categorized into four groups (<25%, 25% to <50%, 50% to <75%, and ≥75%). Odds ratios (ORs) were estimated by fitting a generalized linear mixed model with prefecture as a random intercept and adjusting for covariates (age, gender, body mass index, smoking and drinking status, and comorbidities). Additional analyses according to age group, gender, and wave of the pandemic were conducted.

**Results:**

We observed 2,312 (4.0%) all-cause in-hospital deaths. All-cause in-hospital mortality increased with higher COVID-19 bed utilization rates at diagnosis (OR for multivariable model 1.35; 95% confidence interval [CI], 1.19–1.54 for 25% to <50%; OR 1.89; 95% CI, 1.66–2.16 for 50 to <75%; OR 2.16; 95% CI, 1.80–2.58 for ≥75%; *P* for trend <0.0001). Stronger associations were noted among the younger population (aged <70 years: OR 3.18; 95% CI, 1.96–5.19) and during the fourth (March 1–June 30, 2021: OR 3.81; 95% CI, 2.13–6.80) and sixth pandemic waves (January 1–June 30, 2022: OR 2.67; 95% CI, 1.68–4.23).

**Conclusion:**

Our results emphasize that preventing hospital bed shortages during outbreaks is an important public health strategy to reduce the associated mortality, particularly when new strains emerge and in younger people.

## INTRODUCTION

The coronavirus disease 2019 (COVID-19) pandemic, which began in late 2019, has become a pandemic of unprecedented scale in modern history, causing more than 7 million deaths as of January 2024.^1^ Across countries, the strain on healthcare systems due to the rapid increase in infections relative to healthcare capacity affected care and potentially the survival of patients with COVID-19.^2^^–^^7^ In Japan, the number of COVID-19-specific beds and patient distribution were managed by designating prefectures as health administrative units, and the hospital bed utilization rate (BUR) at the prefectural level was used as a key indicator to assess healthcare system strain and implement proactive intervention, such as the state of emergency.^8^ The state of emergency in Japan was a local government declaration aimed at curbing COVID-19 spread through voluntary compliance, including requests for residents to stay home, businesses to shorten hours or close, event limitations, promoting telework, and so on.^8^

Previous studies from countries such as the United States, United Kingdom, Canada, and Sweden reported that higher COVID-19 bed utilization rates of general wards and of the intensive care unit (ICU) were associated with a higher risk of all-cause mortality in the short term.^2^^–^^7^ A previous study in the United States reported a significant increase in all-cause mortality when COVID-19 ICU demand exceeded 50%.^2^ Another United States study estimated that within two weeks, ICU bed use at 75% capacity would result in 12,000 excess deaths, and at over 100% capacity, 80,000 excess deaths.^7^ However, existing studies were limited to COVID-19 bed utilization during the early stages of the pandemic (up to July 2021),^2^^–^^7^ before the emergence of major new variants and new treatment options. Because healthcare system strain varied largely depending on the type of dominant variants and available treatment strategies for COVID-19, the effect of COVID-19-specific hospital BUR on mortality could differ depending on the specific wave of the pandemic.^9^ In addition, there is little evidence on which population is most affected by healthcare strain, emphasizing the need to design strategies that address the unique needs of different populations during future pandemics.

Therefore, this study examined the association between COVID-19-specific prefectural BUR (which represents BUR by hospital administrative units) and all-cause in-hospital mortality over the first 3 years of the COVID-19 pandemic in Japan (ie, May 2020 to November 2022), while accounting for prefectural clustering using a generalized linear mixed model and adjusting for covariates, such as pre-existing health conditions, vaccination status, smoking and alcohol drinking status, and severity on admission. In addition, we explored whether the impact differed by age, gender, and pandemic wave.

## METHODS

### Data source: COVID-19 Registry Japan

This study used data from the COVID-19 Registry Japan (COVIREGI-JP), a nationwide registry of hospitalized COVID-19 patients, involving 967 public and private hospitals in all prefectures across Japan.^10^ The details of the COVIREJI-JP, including the representativeness of the study, have been described elsewhere.^10^^,^^11^ In brief, COVIREGI-JP collected data from March 17, 2020, to March 31, 2024, mainly from acute healthcare facilities that voluntarily participated in the registry. Patients with a positive SARS-CoV-2 test who received inpatient care for COVID-19 were enrolled.^12^ Patients registered in COVIREGI-JP accounted for approximately 1% of the total number of COVID-19 infections according to national data, with higher coverage during the earlier phase of the pandemic (eg, 32.7% of hospitalized cases in the first wave, January 1 to May 31, 2020) and lower coverage in later waves (eg, less than 1% after the fourth wave, corresponding to April 1 to June 30, 2021). Research collaborators at each facility provided patient demographics, symptoms, and clinical data on admission, clinical course, and discharge outcomes. Standardized COVID-19 care was provided in every prefecture according to the national guidelines,^13^ and all medical expenses related to testing and treatment of COVID-19 were fully subsidized by the government up to May 7, 2023.^14^ The data were managed using the Research Electronic Data Capture (REDCap), a secure web-based application.^15^

### Study population

This study included COVID-19 patients from the COVIREGI-JP who were registered between May 1, 2020, and November 30, 2022, with confirmed data as of December 1, 2022. Of the 64,239 registered participants, we excluded those who lacked information on the date of their first hospital visit (*n* = 1,518), hospital location (*n* = 1,661), or discharge outcome (*n* = 2,712); those diagnosed with COVID-19 after 15 days in hospital (*n* = 166) owing to potential nosocomial infection; and those with missing age data (*n* = 7). Consequently, 58,175 participants were included in this study.

### Outcome: all-cause in-hospital mortality

All-cause in-hospital mortality data were obtained from outcome records at hospital discharge in the COVIREGI-JP database. Other possible outcomes included discharge home, transfer to another medical institution, transfer to a quarantine facility, and admission to a care facility.

### Exposure: COVID-19-specific prefectural hospital bed utilization rate

**Figure 1.**
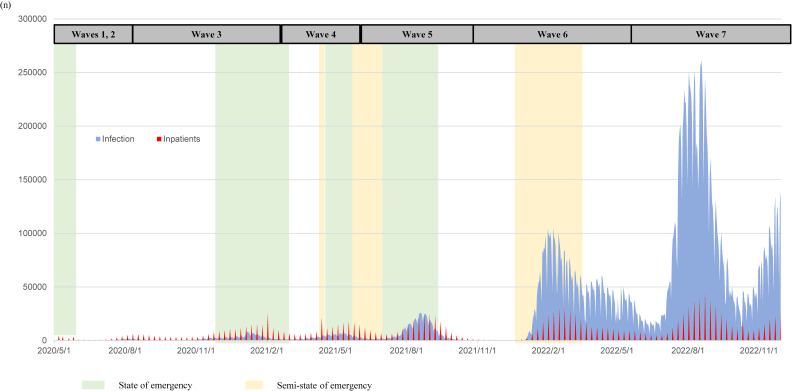
The number of weekly COVID-19 infections and COVID-19 inpatients

The allocation of healthcare resources, including hospital bed capacity and patient distribution, was managed by each prefecture and reported to the Ministry of Health, Labour and Welfare (MHLW). MHLW reported weekly infections and inpatient numbers (Figure 1).^16^ As an indicator of hospital capacity for COVID-19 treatment, we obtained COVID-19-specific prefectural hospital BUR for each participant’s first hospital visit date because there have been cases of delayed hospitalizations when BUR was high. The COVID-19-specific prefectural hospital BUR for each participant was calculated using the following formula: [Number of COVID-19 inpatients in a given prefecture/number of COVID-19-specific beds in that prefecture]. The number of inpatients and COVID-19-specific beds were obtained from the weekly reports of COVID-19 surveillance by the MHLW, Japan.^16^ In order to examine a possible non-linear association, we grouped participants into four categories based on hospital BUR by prefecture as follows: <25%, 25% to <50%, 50% to <75% and ≥75%. In addition, as lifting the state of emergency was considered when the BUR fell below 20%, we analyzed using a cutoff of 20% as follows: <20%, 20% to <50%, 50% to <75%, and ≥75%.

### Covariates

We adjusted for the following factors collected on admission as covariates based on the previous research related to COVID-19 mortality: age, gender (male, female, or other), body mass index (BMI) on admission, smoking status (never smoker, former smoker, current smoker or unknown), alcohol intake (never drinker, current light to moderate drinker, current heavy drinker, or unknown).^17^ We also adjusted for Charlson comorbidity index (CCI) score (0 to 37 points), used to indicate the severity of comorbidities; scores were divided into four categories: low (0 points), medium (1–2 points), high (3–4 points), very high (5 or over).^12^

### Statistical analysis

We first conducted a descriptive analysis of the background characteristics of the participants and assigned them to four groups according to the COVID-19-specific prefectural hospital BUR at their first visit. We then calculated the odds ratios (ORs) with 95% confidence intervals (CIs) for all-cause in-hospital mortality by the level of COVID-19-specific prefectural hospital BUR, using a generalized linear mixed model with prefecture as a random intercept to account for clustering by prefecture. A crude model (model 0), a model adjusted for age and gender (model 1), and a model further adjusted for BMI, drinking status, smoking status, and CCI score (model 2) were examined. Trend tests were performed across the categories. Additionally, since age group and gender were associated with the COVID-19 mortality rate in previous studies,^17^^–^^19^ we repeated the analysis by stratifying by age group (ie, <70, 70–79, and ≥80 years) and gender. Furthermore, to examine whether the associations differed according to the pandemic wave, we repeated the analysis by stratifying the data according to various waves of the pandemic. The study period was divided into six phases based on COVID-19 trends and the dominant variants in Japan: 1) the wild-type dominant early phase (May 1–September 30, 2020), covering the first and second waves in Japan; 2) the wild-type dominant late phase (October 1, 2020–February 28, 2021), the third wave; 3) the alpha variant dominant phase (March 1–June 30, 2021), the fourth wave; 4) the delta variant dominant phase (July 1–December 31, 2021), the fifth wave; 5) the early omicron dominant phase (January 1–June 30, 2022), the sixth wave; and 6) the late omicron dominant phase (July 1–November 30, 2022), the seventh wave. For sensitivity analysis, we further adjusted for the National Early Warning Score (NEWS)^20^ on admission as an indicator of the severity of a patient’s illness. The score was calculated based on the following six physiological parameters: respiratory rate, oxygen saturation, body temperature, blood pressure, heart rate, and level of consciousness. Scores ranged from 0 to 20 points and were divided into three categories: low (0–4 points), medium (5–6 points), and high (>7 points). We also conducted an analysis by adding vaccination status as a covariate in model 2. Vaccination status data collection for COVIREJI began on May 20, 2021, resulting in missing data for vaccinations administered prior to this date, with a missing rate of 66.8% for the study period. However, considering that the vaccination campaign in Japan started in May 2021 for adults aged ≥65 years and in June 2021 for younger populations aged ≥16 years,^21^ we defined vaccination status accordingly. If vaccination information was missing for individuals aged ≥65 years, those registered in the COVID-19 registry before May 2021 were considered unvaccinated, whereas those registered after May 2021 were classified as having an unknown vaccination status. Similarly, for individuals aged <65 years, those registered before June 2021 were considered unvaccinated, whereas those registered after June 2021 were classified as having an unknown vaccination status if vaccination information was missing. Based on this definition, the missing vaccination status rate in this study was reduced to 8.3%, and vaccination status was included as a covariate in the analysis. We used SAS 9.4 (SAS Institute Inc., Cary, NC, USA) for all statistical analyses. All statistical tests were two-tailed, and statistical significance was set at *P* < 0.05.

### Missing data

The proportion of missing data ranged from 0% for age to 16.7% for BMI, except for the NEWS (77.4%), which was used for our sensitivity analysis. Missing data for covariates were imputed using multiple imputations, creating 10 datasets with simulated values from Gibbs sampling, pooled using Rubin’s rules.^22^ The imputed results were broadly similar to those obtained using the observed sample.

## RESULTS

Of the 58,175 participants (mean age: 55.9 years; 55.9% male) included in our study, 16,882 (29.0%) were classified into the <25% BUR category, 23,139 (39.8%) into the 25 to <50% BUR category, 14,681 (25.2%) into the 50 to <75% BUR category, and 3,473 (6.0%) into the ≥75% BUR category according to the COVID-19-specific prefectural hospital BUR (Table 1). The participants in the higher BUR categories were, on average, older and had higher BMI, CCI score, and NEWS; however, the gender ratio did not differ significantly across groups. The proportions of current smokers and heavy drinkers were lower in the higher BUR group. Patient characteristics according to the pandemic wave are shown in eTable 1.

**Table 1.** Patient characteristics (mean values and percentages) according to COVID-19-specific prefectural BUR

Bed utilization rate,^a^ % (*n* = 58,175)	<25%	25% to <50%	50% to <75%	≥75%	*P* value
Number of subjects	16,882	23,139	14,681	3,473	
Age, mean (SD), years	50.8 (24.0)	55.6 (23.4)	60.9 (227)	62.9 (22.2)	<0.0001
<70 years	73.6	66.7	56.9	53.8	
70–79 years	12.5	14.9	18.5	18.6	
≥80 years	14.0	18.4	24.6	27.6	
Male, %	56.3	55.4	56.3	56.5	0.26
Smoking status					
Current smoker, %	16.7	14.7	12.1	12.2	<0.0001
Former smoker, %	19.0	20.1	21.7	20.6	
Never smoker, %	51.0	50.2	49.2	48.0	
Unknown, %	13.4	15.0	17.1	19.1	
Drinking status					
Current heavy drinker, %	6.2	5.4	3.9	3.9	<0.0001
Current light to moderate drinker, %	31.9	29.7	27.0	24.5	
Never drinker, %	41.4	41.5	44.2	46.1	
Unknown, %	20.5	23.4	24.9	25.6	
Body mass index, mean (SD), kg/m^2^	23.5 (4.9)	23.8 (4.9)	23.7 (4.9)	23.8 (5.0)	<0.0001
<18.5, %	12.9	11.8	12.2	12.7	
18.5 to <25.0, %	54.2	52.4	52.0	51.5	
≥25.0, %	32.9	35.8	35.8	35.8	
Charlson comorbidity index score^b^					
Low (0 points), %	67.6	62.5	52.9	48.0	<0.0001
Medium (1–2 points), %	25.4	29.2	35.9	37.5	
High (3–4 points), %	5.0	5.9	8.2	10.8	
Very high (over 5 points), %	2.0	2.4	3.0	3.7	
NEWS on admission^c^					
Low (0–4 points), %	67.3	64.4	56.9	51.0	<0.0001
Medium (5–6 points), %	20.0	21.1	23.5	25.0	
High (over 7 points), %	12.8	14.6	19.7	24.0	
Pandemic wave					
Waves 1 and 2, *May 1 to September 30, 2020*	28.7	19.5	7.6	5.0	<0.0001
Wave 3, *October 1, 2020 to February 28, 2021*	26.5	32.5	41.3	20.1	
Wave 4, *March 1 to June 30, 2021*	6.7	11.2	9.2	18.5	
Wave 5, *July 1 to December 31, 2021*	14.8	17.2	19.2	17.9	
Wave 6, *January 1 to June 30, 2022*	17.9	14.4	15.2	16.2	
Wave 7, *July 1 to November 30, 2022*	5.3	5.2	7.6	22.3	

BUR, bed utilization rate; COVID-19, coronavirus disease 2019; IQR, interquartile range; SD, standard deviation.

^a^The ratio of the number of inpatients to the number of beds, %.

^b^A measure of medical comorbidity in which increasing values indicate a greater comorbidity burden.

^c^This score indicates the degree of illness of a patient and the need for prompt critical care interventions.

In our study, there were 2,312 (4.0%) all-cause in-hospital deaths among COVID-19 inpatients. In the crude model, the association between the risk of all-cause in-hospital mortality and COVID-19-specific prefectural hospital BUR was statistically significant. Compared with the <25% group, the OR was 3.10 (95% CI, 2.60–3.69) for the ≥75% group, 2.52 (95% CI, 2.22–2.86) for the 50% to <75% group, and 1.51 (95% CI, 1.34–1.72) for the 25% to <50% group (*P* for trend <0.0001; Table 2, model 0). The risks were attenuated after adjusting for age and gender but remained statistically significant with OR of 1.89 (95% CI, 1.57–2.27) for the ≥75% group, 1.61 (95% CI, 1.41–1.84) for the 50% to <75% group, and 1.21 (95% CI, 1.06–1.38) for the 25% to <50% group compared with the <25% group (*P* for trend <0.0001; Table 2, model 1). In our multivariable adjusted model, the risks remained significant for all groups compared with the <25% group, with OR of 2.16 (95% CI, 1.80–2.58) for the ≥75% group, 1.89 (95% CI, 1.66–2.16) for the 50% to <75% group, and 1.35 (95% CI, 1.19–1.54) for the 25% to <50% group (*P* for trend <0.0001; Table 2, model 2). When continuous variables were incorporated into model 2, each 1% increase in BUR was associated with a 1.008-fold increase in the odds of in-hospital mortality: OR 1.008 (95% CI, 1.006–1.009, *P* for trend <0.0001). The positive association between COVID-19-specific prefectural BUR and all-cause in-hospital mortality among patients with COVID-19 was stronger when the reference group was changed to 20% (eTable 2). Additionally, the results remained robust even after adjusting for vaccination status in model 2 (eTable 3).

**Table 2.** Associations between COVID-19-specific prefectural BUR and risk of all-cause in-hospital mortality for multivariable model

Bed utilization rate,^a^ %	<25%	25% to <50%	50% to <75%	≥75%	*P* for linear trend
Number at risk	16,882	23,139	14,681	3,473	
Number of deaths	410	801	862	239	
Crude model	1	1.51 (1.34–1.72)	2.52 (2.22–2.86)	3.10 (2.60–3.69)	<0.0001
Model 1: Age and gender-adjusted OR, (95% CI)	1	1.21 (1.06–1.38)	1.61 (1.41–1.84)	1.89 (1.57–2.27)	<0.0001
Model 2: Multivariable OR, (95% CI)^b^	1	1.35 (1.19–1.54)	1.89 (1.66–2.16)	2.16 (1.80–2.58)	<0.0001

BUR, bed utilization rate; CI, confidence interval; COVID-19, coronavirus disease 2019; OR, odds ratio.

^a^The ratio of the number of inpatients to the number of beds, %.

^b^Adjusted further for body mass index, drinking status, smoking status, Charlson comorbidity index score.

Further analysis stratified by age group revealed that the effect of prefectural hospital BUR on the risk of all-cause in-hospital mortality was most pronounced for those aged <70 years compared with older age groups (ie, 70–79 years, ≥80 years) in the multivariable-adjusted model. For example, for the ≥75% group, the OR for those aged >70 years was 3.18 (95% CI, 1.96–5.19), whereas the OR for those aged 70 to 79 years was 2.40 (95% CI, 1.69–3.40), and 1.35 (95% CI, 1.06–1.73) for those aged ≥80 years (Table 3, model 2). The ratio of those categorized as having a “high” NEWS on admission was 8.7% for those aged <70 years, 3.0% for those aged 70 to 79 years, and 4.2% for those aged ≥80 years.

**Table 3.** Associations between BUR and in-hospital mortality by age, gender and pandemic wave for model 2

Bed utilization rate,^a^ %	<25%	25% to <50%	50% to <75%	≥75%	*P* for linear trend	*P* for interaction
***Age***						0.0011
*<70 years*						
Number at risk	12,423	15,428	8,354	1,869		
Number of deaths	50	114	125	30		
Multivariable OR (95% CI)^b^	1	1.72 (1.21–2.44)	2.99 (2.10–4.25)	3.18 (1.96–5.19)	<0.0001	
*70*–*79 years*						
Number at risk	2,103	3,444	2,715	647		
Number of deaths	101	190	202	71		
Multivariable OR (95% CI)^b^	1	1.23 (0.95–1.61)	1.64 (1.25–2.14)	2.40 (1.69–3.40)	<0.0001	
*≥80 years*						
Number at risk	2,356	4,267	3,612	957		
Number of deaths	259	497	535	138		
Multivariable OR (95% CI)^b^	1	1.08 (0.91–1.27)	1.33 (1.12–1.57)	1.35 (1.06–1.73)	0.0020	
***Gender*** ^c^						
*Male*						0.71
Number at risk	9,497	12,829	8,263	1,962		
Number of deaths	247	490	524	149		
Multivariable OR (95% CI)^b^	1	1.25 (1.05–1.48)	1.53 (1.29–1.82)	1.70 (1.34–2.16)	<0.001	
*Female*						
Number at risk	7,379	10,298	6,412	1,511		
Number of deaths	163	311	338	90		
Multivariable OR (95% CI)^b^	1	1.17 (0.95–1.44)	1.65 (1.33–2.04)	1.93 (1.43–2.59)	<0.001	
***Pandemic wave***						
*Waves 1 and 2 (May 1 to September 30, 2020)*						<0.0001
Number at risk	4,846	4,501	1,116	175		
Number of deaths	104	135	36	3		
Multivariable OR (95% CI)^b^	1	1.31 (0.93–1.83)	1.31 (0.82–2.10)	0.92 (0.25–3.37)	0.38	
*Wave 3 (October 1, 2020 to February 28, 2021)*						
Number at risk	4,480	7,519	6,059	698		
Number of deaths	125	337	441	53		
Multivariable OR (95% CI)^b^	1	1.14 (0.90–1.43)	1.42 (1.13–1.80)	1.48 (1.00–2.18)	0.0076	
*Wave 4 (March 1 to June 30, 2021)*						
Number at risk	1,134	2,591	1,357	642		
Number of deaths	45	87	79	62		
Multivariable OR (95% CI)^b^	1	1.01 (0.66–1.56)	1.53 (0.97–2.43)	3.81 (2.13–6.80)	<0.0001	
*Wave 5 (July 1 to December 31, 2021)*						
Number at risk	2,502	3,984	2,813	620		
Number of deaths	34	87	117	25		
Multivariable OR (95% CI)^b^	1	1.38 (0.89–2.15)	2.27 (1.47–3.52)	1.96 (1.06–3.64)	0.0011	
*Wave 6 (January 1 to June 30, 2022)*						
Number at risk	3,023	3,340	2,225	563		
Number of deaths	68	114	124	45		
Multivariable OR (95% CI)^b^	1	1.29 (0.92–1.79)	1.67 (1.18–2.35)	2.67 (1.68–4.23)	0.0004	
*Wave 7 (July 1 to November 30, 2022)*						
Number at risk	897	1,204	1,111	775		
Number of deaths	34	41	65	51		
Multivariable OR (95% CI)^b^	1	0.87 (0.53–1.42)	1.27 (0.81–2.01)	1.34 (0.81–2.21)	0.20	

BUR, bed utilization rate; CI, confidence interval; OR, odds ratio.

^a^The ratio of the number of inpatients to the number of beds, %.

^b^Adjusted further for body mass index, drinking status, smoking status, and Charlson comorbidity index score.

^c^24 patients with “Other” gender were excluded from the analysis.

Our analysis stratified by pandemic waves showed that the association between COVID-19-specific prefectural hospital BUR and all-cause in-hospital mortality differed by pandemic wave (Table 3). In the multivariable model, there was no association between COVID-19-specific prefectural hospital BUR and all-cause in-hospital mortality in the first and second waves when the number of COVID-19 patients was relatively small (the maximum daily number of cases was 1,597 cases per day during this period), or in the seventh wave when the omicron variant was the dominant type for the second time. However, there was a significant association during the third, fourth, fifth, and sixth waves, with the association being stronger in the fourth and sixth waves (the OR for the ≥75% group was 3.81; 95% CI, 2.13–6.80 for the fourth wave and 2.67; 95% CI, 1.68–4.23 for the sixth wave).

Our sensitivity analysis further adjusting for NEWS attenuated the association, particularly for the ≥75% group (eTable 4). Nevertheless, the association between COVID-19-specific hospital BUR and all-cause in-hospital mortality remained significant even after adjusting for the NEWS.

## DISCUSSION

Our large-scale nationwide study over 3 years found a positive dose-response association between COVID-19-specific prefectural BUR and all-cause in-hospital mortality. Compared with the BUR <25% group, the odds were 1.9-fold higher when the BUR was 50% to <75% and 2.2-fold higher when it exceeded 75%, even after adjusting for various patient characteristics. Additional analysis revealed that this association was greater for patients aged <70 years than for older age groups and was more pronounced during the fourth and sixth waves of the pandemic, when new variants emerged.

The observed increase in all-cause in-hospital mortality with higher prefectural hospital BUR was consistent with the results of studies conducted in Western countries,^2^^–^^7^ despite the differences in each country’s infection situation or pandemic response. A previous study in the United States reported a significant increase in all-cause mortality when COVID-19 ICU demand was more than 50%.^2^ In line with the results of the previous study, the association between BUR and mortality was most evident when the BUR exceeded 50%. Previous research has examined COVID-19 mortality based on the BUR for ICU,^2^ whereas this study focused on the overall BUR, including both ICU and general hospital beds. Therefore, direct comparison was difficult. However, both previous studies and ours highlight the effect of healthcare capacity constraints on in-hospital mortality and emphasize the importance of monitoring BUR to inform timely public health interventions. By incorporating the overall hospital BUR, this study complements previous research by offering a more comprehensive perspective on the broader impact of healthcare system strain.

Furthermore, the association between COVID-19-specific prefectural hospital BUR and all-cause in-hospital mortality remained significant in our sensitivity analysis, which was further adjusted for severity on admission using the NEWS and vaccination status. Notably, adjusting for NEWS attenuated this association; however, the association was not fully explained. These findings suggest that, although higher severity on admission may partly contribute to the observed association between COVID-19-specific prefectural hospital BUR and all-cause in-hospital mortality, other factors related to healthcare system strain, such as reduced staff availability and resource shortages, may also play a significant role. The results of the present study suggest that utilizing COVID-19-specific prefectural BUR in proactive interventions, such as the declaration of a state of emergency when the BUR exceeds 50%, could be a useful indicator to avoid healthcare system overload. Additionally, the findings highlight the importance of strategies to optimize bed utilization during a pandemic, which should be addressed at both the hospital and administrative levels. A system was established during the pandemic to address the need for continued inpatient care for patients recovering from COVID-19, designating certain hospitals to treat patients in the post-acute phase. Prefectural governments coordinated admissions under the Infectious Diseases Act,^23^ and in some prefectures, they facilitated transfers for patients needing long-term care. For such systems to function effectively in future public health emergencies, it is crucial to establish a centralized coordination mechanism to oversee patient transfers and optimize resource allocation. Implementing clear referral pathways and enhancing coordination among healthcare facilities could further streamline hospital discharge, contributing to shorter lengths of stay, improved bed turnover, and more efficient use of healthcare resources.

In our study, the risk of all-cause in-hospital mortality was higher in patients aged <70 years than in older patients, indicating that the effect of increased hospital BUR was more pronounced in the younger age group. One possible explanation is that younger COVID-19 patients were prioritized for home care, reserving hospitalization for high-risk groups, such as older individuals and those with underlying health conditions,^13^ so they may have been hospitalized only when their condition had already worsened. Supporting this hypothesis, COVID-19 severity on hospitalization was higher for patients aged <70 years compared with those aged ≥70 years: the ratio of those categorized as having a “high” NEWS on admission was 8.7% for those aged under 70 years, 3.0% for those aged 70 to 79 years, and 4.2% for those aged 80 years and older. Our sensitivity analysis, further adjusted for NEWS, revealed that the association between COVID-19-specific prefectural BUR and the risk of all-cause in-hospital mortality was attenuated, particularly in those aged <70 years. This indicates that younger individuals tended to have higher severity upon admission and were more susceptible to the increased mortality risk associated with higher BUR.

The impact of increased hospital BUR on all-cause in-hospital mortality varied according to pandemic wave. The highest risk of all-cause mortality when the BUR was ≥75% was observed in the fourth and sixth waves, when new variants emerged. In the fourth wave, the alpha variant emerged, which had high pathogenicity and transmissibility compared with the conventional strain.^24^ Furthermore, vaccination coverage had just begun and remained low in Japan during this period.^21^ This led to a sudden surge in the number of infections during the fourth wave. Similarly, during the sixth wave, a new variant (omicron) emerged that had a lower severity but much higher infection rates.^25^ The rapid rise in infections not only increased hospitalization needs but also caused healthcare worker shortages due to quarantine, which included those infected and those who had close contact with a patient.^26^ In the early phase of the pandemic, including the first and second waves, non-COVID-19 care was scaled down, allowing staff to be reassigned to focus on COVID-19 treatment. However, in the later phase of the pandemic, including the sixth and seventh waves, hospitals had to balance non-COVID-19 care with COVID-19 treatment. Furthermore, as observed in our study, hospitalized patients during the sixth and seventh waves of the pandemic were older, had more severe symptoms on admission, and had more complications than hospitalized patients during earlier phases, indicating greater care needs in later waves. The stronger association between BUR and mortality observed in the later phase may be attributed to the increased impact of staff shortages on healthcare system during this period, as previously reported in the United States.^27^ Our findings suggest that when a new variant emerges the effect of a high BUR on the mortality of COVID-19 patients may become more pronounced and thus, it is necessary to restructure healthcare strategies in accordance with the characteristics of the variant.^28^

The present study has several strengths, including its use of data from a nationwide large-scale registry covering the first 3 years of the pandemic. This study also considers bed capacity on a prefectural basis, employing robust statistical methods to account for clustering effects within each prefecture. The following limitations warrant further discussion. First, although this study is based on the largest national registry of COVID-19 patients covering all prefectures in Japan, selection bias may have occurred because of the nature of the registry study, as described previously.^11^ Second, although we carefully adjusted for known lifestyle- and comorbidity-related risk factors, other residual confounding factors, such as socioeconomic status or cohabitation status, may exist. In addition, clarifying the underlying mechanism by which COVID-19 bed utilization leads to increased in-hospital mortality can offer additional evidence to mitigate its influence on the survival outcomes of COVID-19 patients during the pandemic. This investigation considers not only bed occupancy rates but also healthcare workforce availability, the quality of care provided, and other pertinent factors.

In conclusion, our large-scale nationwide study of COVID-19 in-hospital patients found a positive dose-response association between the COVID-19-specific prefectural bed utilization rate and the risk of all-cause in-hospital mortality. The pronounced risks among the younger age group and during the fourth and sixth waves in Japan, when the alpha and omicron variants emerged, further highlight the importance of preparedness in mitigating the effect of bed shortages on mortality.

## SUPPLEMENTARY MATERIAL

eTable 1. Patient characteristics (mean values and percentages) according to the pandemic wave

eTable 2. Associations between COVID-19-specific prefectural bed utilization rate and risk of all-cause in-hospital mortality with a 20% cutoff

eTable 3. Associations between COVID-19-specific prefectural BUR and risk of all-cause in-hospital mortality for multivariable model

eTable 4. Sensitivity analysis on the association between COVID-19-specific prefectural bed utilization rate categories and the risk of all-cause of mortality adjusted further for NEWS score on admission
