# Supplementary material for: COVID-19-specific Prefectural Hospital Bed Utilization Rate and In-hospital Mortality Among COVID-19 Patients Throughout the First 3 Years of the Pandemic in Japan
Source: J Epidemiol. 2025 Sep 5;35(9):402–9. doi: 10.2188/jea.JE20240395 (PMC12358257; doi:10.2188/jea.JE20240395)
Supplement: Supplementary file 1 [file je-35-402-s001.pdf]

eTable 1. Patient characteristics (mean values and percentages) according to the pandemic wave

	Waves 1 and 2 *(1 May 2020 to 30 Sep 2020)*	Wave 3 (*1 Oct 2020 to 28 Feb 2021*)	Wave 4 (*1 Mar 2021 to 30 Jun 2021*)	Wave 5 (*1 Jul 2021 to 31 Dec 2021*)	Wave 6 (*1 Jan 2022 to 30 Jun 2022*)	Wave 7 (*1 Jul 2022 to 30 Nov 2022*)
Number of patients	10,638	18,756	5,724	9,919	9,151	3,987
Age, mean (SD), years	49.7 (22.6)	60.0 (21.7)	57.3 (21.7)	47.7 (19.8)	59.5 (27.3)	64.1 (28.6)
<70 years, %	76.3	60.2	65.4	86.3	51.8	40.6
70–79 years, %	11.9	19.0	18.1	7.1	17.8	18.2
≥80 years, %	11.8	20.8	16.5	6.7	30.4	41.2
Male, %	57.5	55.8	57.2	60.9	51.2	49.4
Smoking status						
Current smoker, %	19.0	12.3	15.2	20.5	10.2	6.4
Former smoker, %	18.2	21.9	19.7	19.2	20.5	20.1
Never smoker, %	47.6	48.0	49.0	50.8	53.8	57.4
Unknown, %	15.1	17.9	16.1	9.5	15.5	16.1
Drinking status, %						
Current heavy drinker, %	8.7	6.1	4.2	4.6	1.9	1.4
Current light to moderate drinker, %	35.3	30.7	30.7	33.5	20.2	16.1
Never drinker, %	33.2	39.2	40.6	40.0	54.0	64.2
Unknown, %	22.8	24.0	24.6	21.9	23.9	18.3

Mean body mass index (SD), kg/m^2	23.7 (4.8)	23.9 (4.7)	24.2 (4.8)	24.6 (5.2)	22.9 (4.9)	22.0 (4.9)
<18.5, %	11.6	10.3	9.0	9.5	17.2	23.7
18.5 to <25.0, %	54.4	53.6	52.8	48.7	53.6	52.7
≥25.0, %	34.0	36.1	38.2	41.8	29.2	23.6
Charlson comorbidity index score[a]						
Low (0 points), %	72.8	58.4	65.4	71.6	46.2	38.6
Medium (1–2 point), %	21.6	33.2	29.1	23.8	37.4	41.1
High (3–4 points), %	4.2	6.3	4.1	3.3	11.4	14.3
Very high (over 5 points), %	1.5	2.0	1.5	1.3	5.0	6.0
NEWS on admission[b]						
Low (0–4 points), %	71.3	64.9	62.1	56.0	60.4	49.5
Medium (5–6 points), %	17.7	20.5	21.9	25.0	23.0	25.3
High (over 7 points), %	11.0	14.7	16.0	19.0	16.6	25.2

NEWS, National Early Warning Score; SD, standard deviation.

[a] A measure of medical comorbidity in which increasing values indicate a greater comorbidity burden.

[b] This score indicates the degree of illness of a patient as well as the need for prompt critical care intervention.

eTable 2. Associations between COVID-19-specific prefectural bed utilization rate and risk of all-cause in-hospital mortality with a 20% cutoff

**Bed utilization rate,[a] %**	**<20%**	**20% to <50%**	**50% to <75%**	**≥75%**	***P* for linear trend**
Number at risk	12,505	27,516	14,681	3,473	
Number of deaths	266	945	862	239	
Model 1: Age and gender-adjusted OR (95% CI)	1	1.32 (1.12–1.52)	1.75 (1.50–2.04)	2.05 (1.68–2.50)	<0.0001
Model 2: Multivariable OR (95% CI)[b]	1	1.50 (1.29–1.73)	2.12 (1.82–2.46)	2.40 (1.98–2.92)	<0.0001

CI, confidence interval; OR, odds ratio.

[a] The ratio of the number of inpatients to the number of beds, %

[b] Adjusted further for body mass index, drinking status, smoking status, Charlson comorbidity index score

eTable 3. Associations between COVID-19-specific prefectural BUR and risk of all-cause in-hospital mortality for multivariable model

**Bed utilization rate,[a] %**	**<25%**	**25% to <50%**	**50% to <75%**	**≥75%**	***P* for linear trend**
Number at risk	16,882	23,139	14,681	3,473	
Number of deaths	410	801	862	239	
Model 3: Multivariable OR, (95% CI)[b]	1	1.32 (1.16–1.50)	1.87 (1.64–2.13)	2.18 (1.82–2.62)	<0.0001

BUR, bed utilization rate, CI, confidence interval; COVID-19, coronavirus disease 2019; OR, odds ratio.
[a] The ratio of the number of inpatients to the number of beds, %
[b] Adjusted further for body mass index, drinking status, smoking status, Charlson comorbidity index score and vaccination status

eTable 4. Sensitivity analysis on the association between COVID-19-specific prefectural bed utilization rate categories and the risk of all-cause of mortality adjusted further for NEWS score on admission

**Bed utilization rate,[a] %**	**<25%**	**25% to <50%**	**50% to <75%**	**≥75%**	***P* for linear trend**	***P* for interaction**
Number at risk	16,882	23,139	14,681	3,473		
Number of deaths	410	801	862	239		
Multivariable OR (95% CI)[b]	1	1.27 (1.11–1.45)	1.63 (1.42–1.86)	1.63 (1.35–1.96)	<0.0001	
Age						0.097
<70 years						
Number at risk	12,423	15,428	8,354	1,869		
Number of deaths	50	114	125	30		
Multivariable OR (95% CI)[b]	1	1.40 (0.98–2.00)	2.11 (1.47–3.03)	1.90 (1.15–3.13)	0.0004	
70–79 years						
Number at risk	2,103	3,444	2,715	647		
Number of deaths	101	190	202	71		
Multivariable OR (95% CI)[b]	1.00	1.22 (0.93–1.60)	1.46 (1.11–1.92)	1.85 (1.29–2.66)	0.0041	
≥80 years						
Number at risk	2,356	4,267	3,612	957		
Number of deaths	259	497	535	138		
Multivariable OR (95% CI)[b]	1.00	1.07 (0.90–1.27)	1.25 (1.05–1.49)	1.15(0.90–1.48)	0.058	
Gender[c]						

Male						0.72
Number at risk	9,497	12,829	8,263	1,962		
Number of deaths	247	490	524	149		
Multivariable OR (95% CI)[b]	1	1.27 (1.07–1.50)	1.58 (1.33–1.88)	1.59 (1.26–2.02)	<0.0001	
Female						
Number at risk	7,379	10,298	6,412	1,511		
Number of deaths	163	311	338	90		
Multivariable OR (95% CI)[b]	1	1.27 (1.04–1.57)	1.69 (1.37–2.09)	1.69 (1.25–2.28)	<0.0001	
Pandemic wave						
Waves 1 and 2 (1 May 2020 to 30 Sep 2020)						<0.0001
Number at risk	4,846	4,501	1,116	175		
Number of deaths	104	135	36	3		
Multivariable OR (95% CI)[b]	1	1.36 (1.00–1.84)	1.39 (0.87–2.20)	0.79 (0.21–2.94)	0.19	
Wave 3 (1 Oct 2020 to 28 Feb 2021)						
Number at risk	4,480	7,519	6,059	698		
Number of deaths	125	337	441	53		
Multivariable OR (95% CI)[b]	1	1.10 (0.87–1.40)	1.33 (1.05–1.67)	1.40 (0.95–2.06)	0.040	
Wave 4 (1 Mar 2021 to 30 Jun 2021)						
Number at risk	1,134	2,591	1,357	642		
Number of deaths	45	87	79	62		
Multivariable OR (95% CI)[b]	1	0.96 (0.61–1.52)	1.23 (0.76–1.99)	2.12 (1.16–3.90)	0.039	

Wave 5 (1 Jul 2021 to 31 Dec 2021)					
Number at risk	2,502	3,984	2,813	620	
Number of deaths	34	87	117	25	
Multivariable OR (95% CI)[b]	1	1.17 (0.74–1.85)	1.75 (1.11–2.75)	1.40 (0.74–2.64)	0.042
Wave 6 (1 Jan 2022 to 30 Jun 2022)					
Number at risk	3,023	3,340	2,225	563	
Number of deaths	68	114	124	45	
Multivariable OR (95% CI)[b]	1	1.21 (0.86–1.70)	1.45 (1.02–2.06)	2.21 (1.38–3.56)	0.0097
Wave 7 (1 Jul 2022 to 30 Nov 2022)					
Number at risk	897	1,204	1,111	775	
Number of deaths	34	41	65	51	
Multivariable OR (95% CI)[b]	1	0.78 (0.47–1.29)	1.20 (0.75–1.92)	1.20 (0.72–2.00)	0.20

CI, confidence interval; COVID-19, coronavirus disease 2019; OR, odds ratio.

[a] The ratio of the number of inpatients to the number of beds, %

[b] Adjusted further for body mass index, drinking status, smoking status, Charlson comorbidity index score, and NEWS score on admission

[c] 24 patients with “Other” gender were excluded from the analysis.
